# Transcriptome-wide analysis of microRNA expression in the malaria mosquito *Anopheles gambiae*

**DOI:** 10.1186/1471-2164-15-557

**Published:** 2014-07-04

**Authors:** Inna Biryukova, Tao Ye, Elena Levashina

**Affiliations:** Department of Vector Biology, Max Planck Institute for Infection Biology, Berlin, 10117 Germany; Microarrays and deep sequencing platform, IGBMC, Illkirch, Cedex, 67404 France

## Abstract

**Background:**

microRNAs (miRNAs) are a highly abundant class of small noncoding regulatory RNAs that post-transcriptionally regulate gene expression in multicellular organisms. miRNAs are involved in a wide range of biological and physiological processes, including the regulation of host immune responses to microbial infections. Small-scale studies of miRNA expression in the malaria mosquito *Anopheles gambiae* have been reported, however no comprehensive analysis of miRNAs has been performed so far.

**Results:**

Using small RNA sequencing, we characterized *de novo A. gambiae* miRNA repertoire expressed in adult sugar- and blood-fed females. We provided transcriptional evidences for 123 miRNAs, including 58 newly identified miRNAs. Out of the newly described miRNAs, 19 miRNAs are homologs to known miRNAs in other insect species and 17 miRNAs share sequence similarity restricted to the seed sequence. The remaining 21 novel miRNAs displayed no obvious sequence homology with known miRNAs. Detailed bioinformatics analysis of the mature miRNAs revealed a sequence variation occurring at their 5’-end and leading to functional seed shifting in more than 5% of miRNAs. We also detected significant sequence heterogeneity at the 3’-ends of the mature miRNAs, mostly due to imprecise processing and post-transcriptional modifications. Comparative analysis of arm-switching events revealed the existence of species-specific production of dominant mature miRNAs induced by blood feeding in mosquitoes. We also identified new conserved and fragmented miRNA clusters and *A. gambiae*-specific miRNA gene duplication. Using miRNA expression profiling, we identified the differentially expressed miRNAs at an early time point after regular blood feeding and after infection with the rodent malaria parasite *Plasmodium berghei*. Significant changes were detected in the expression levels of 4 miRNAs in blood-fed mosquitoes, whereas 6 miRNAs were significantly upregulated after *P. berghei* infection.

**Conclusions:**

In the current study, we performed the first systematic analysis of miRNAs in *A. gambiae*. We provided new insights on mature miRNA sequence diversity and functional shifts in the mosquito miRNA evolution. We identified a set of the differentially expressed miRNAs that respond to normal and infectious blood meals. The extended set of *Anopheles* miRNAs and their isoforms provides a basis for further experimental studies of miRNA expression patterns and biological functions in *A. gambiae*.

**Electronic supplementary material:**

The online version of this article (doi:10.1186/1471-2164-15-557) contains supplementary material, which is available to authorized users.

## Background

Hematophagous females of *A. gambiae* require animal blood for successful reproduction. Sequential blood intake is the main route for transmission of the protozoan parasite *Plasmodium*, the causative agent of malaria. The ability of *Plasmodium* parasites to establish infection in the vector mosquito can be compromised by many factors, including mosquito innate immune responses and factors derived from the blood of the human host [1, 2]. A better understanding of regulatory circuits and mechanisms that regulate mosquito biology and contribute to vector resistance to *Plasmodium* parasites is urgently needed to curb malaria transmission.

miRNAs are approximately 22 nucleotides RNAs that regulate and influence a wide range of biological and physiological processes in metazoans and plants, playing instructive role throughout development and conferring robustness to gene expression [3, 4]. In insects, the miRNA pathway has been best characterized in the fruit fly, *D. melanogaster*. The canonical miRNA biogenesis starts from transcription of endogenous primary miRNA transcripts typically produced by RNA polymerase lI. The primary transcripts frequently contain multiple miRNA hairpin precursors, which are processed by the nuclear heterodimer DGCR-8 and the RNAse III enzyme, Drosha. The released ~55-70 nt cleavage product, called a pre-miRNA hairpin is exported to the cytoplasm. Once in the cytoplasm, the pre-miRNA is processed by another RNAse III enzyme, Dicer-1, yielding ~22 nt small RNA duplexes [3, 5]. In *Drosophila*, one of the strands (called the “guiding”) is preferentially incorporated into an effector miRNA-induced silencing complex (miRISC) containing the Argonaute-family protein AGO1. The other (“passenger”) strand is sorted into small interfering RNAs, siRISC complex with AGO2 effector protein [6]. A number of alternative Drosha/Dicer-independent pathways producing functional miRNAs have been reported [7]. However, regardless of the miRNA biogenesis diversity, the stability and silencing activity of mature miRNA predominantly require AGO effector proteins [7–9]. A comparative phylogenetic analysis of small regulatory RNA pathways revealed that major components of miRNA biogenesis and AGO effector proteins are conserved between *A. gambiae* and *D. melanogaster* [10]. It has been shown that siRNA-mediated silencing in *A. gambiae* requires AGO2 [11], however no evidence of functional association between AGO1 and miRNAs in *A. gambiae* has been demonstrated so far.

miRNAs act as antisense guide for the miRISC complex to recognize target protein coding and non-coding RNAs. miRNA-target interactions are based on Watson-Crick base pairing between the miRNA seed region (nucleotides 2–8 relative to its 5’-end) and target RNA [3, 12], typically leading to mRNA destabilization and translational repression [13]. miRNA expression levels and patterns rely on both steps of their biogenesis: transcription and processing, and are tightly regulated temporally and spatially during development [14, 15]. The predominant mature miRNA can be produced from both the 5’- and 3’-arms of pre-miRNA hairpins. Selection of the functional arm, the precision of miRNA processing and post-transcriptional modifications play critical roles in the refining and diversifying of mature miRNA sequence and eventual functional activity [16, 17]. Post-transcriptional modifications at the 3’-end, mostly non-template directed adenylation and uridylation, alter miRNA activity and stability by regulating either processing by Dicer-1 or incorporation into miRISC [18–21].

It has recently been reported that miRNAs can sense biotic stresses operating as an integral part of host immune responses to microbial infections, caused by viral, bacterial and *Apicomplexan* pathogens [22–24]. Interestingly, in *A. gambiae* the upstream control regions of the major components of miRNA biogenesis, *Drosha*, *Dcr-1* and *Ago-1* are enriched in the potential binding sites for NF-kappaB-related transcription factors [10], presumably providing a link between the miRNA pathway and immune responses. Moreover, RNA silencing of *Ago-1* and *Dcr-1* in *A. gambiae* mosquitoes resulted in the increased survival of the rodent malaria parasite *P. berghei* [25]. Furthermore, a recent study using ribosome profiling in *A. gambiae* infected with the human malaria parasite *P. falciparum* revealed an enhanced association of ~35 mosquito immune-related transcripts, including two components of the miRNA pathway *Dcr-1* and *Drosha* with polyribosomes [26].

In this study, using small RNA sequencing we performed the first systematic analysis of *A. gambiae* miRNAs in adult sugar- and blood-fed females. We provided transcriptional evidence for a wide diversity of mature miRNAs and their isoforms. Our analysis revealed significant sequence variations among mature miRNAs at their 3’-ends, mostly due to imprecise processing during their biogenesis and post-transcriptional modifications. Furthermore, our extended set of *Anopheles* miRNAs allows us to analyze their expression at an early time point after regular and infectious blood feeding. As a result, we identified 6 differentially expressed *Anopheles* miRNAs associated with the rodent malaria parasite *P. berghei* infections.

## Results

### *A. gambiae*small RNA sequencing

**Figure 1:**
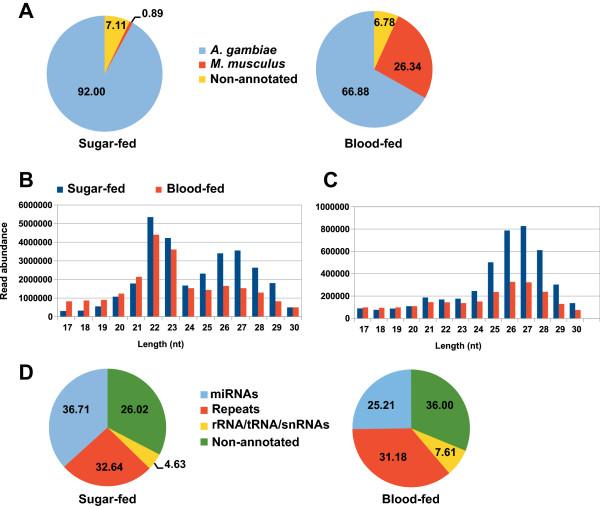
**Sequencing summary of small RNAs in** ***A. gambiae*** **. (A)** Read frequency for all sequences assigned to *A. gambiae* and *M. musculus* genomes in sugar and blood-fed mosquitoes. Size distribution for the total number of sequence reads **(B)** and for unique sequence reads **(C)**. The abundance of the reads between 17 and 30 nt from sugar and blood-fed mosquitoes are as indicated. **(D)** Read frequency for all sequences of small RNA reads. The identity and abundance of small RNA reads in sugar and blood-fed mosquitoes are as indicated.

The majority of the originally annotated *Anopheles* miRNAs have been identified by sequence similarity with *D. melanogaster* miRNA orthologs using sequence and structure alignment [27, 28]. In addition, a number of mosquito specific miRNAs identified in the closely related species, *A. stephensi, Cx. quinquefasciatus* and *Ae. aegypti* by cloning [29] and deep sequencing [30, 31] has been shown to be conserved in *A. gambiae*. Among 67 *Anopheles* miRNAs reported in miRBase, less than a half were experimentally validated [25, 29, 30]. In order to identify and characterize *de novo* miRNAs in *A. gambiae*, we constructed small RNA libraries from sugar and blood-fed *A. gambiae* G3 adult females, including fecund females. Two independent libraries (biological replicates) were prepared and independently sequenced using the Illumina high-throughput sequencing platform, yielding a total of ~67.1 million sequence reads. We observed a significant correlation between two independent libraries sequencing results (R^2^ = 0.95 for sugar-fed and R^2^ = 0.96 for blood-fed samples). Since the genome of the G3 strain has not been sequenced, the *A. gambiae* PEST strain genome was used as a reference. After adapter trimming and filtering out ambiguous reads, a total of ~57.3 million sequence reads were obtained and ~46.2 million sequence reads were aligned. Around 92.00% and 66.88% of the total reads were mapped to the reference genome in sugar and blood-fed mosquitoes, respectively (Figure 1A). Less than 1% of sequence reads were mapped to the *M. musculus* genome in sugar-fed mosquitoes, while in blood-fed samples ~26.34% of sequence reads derived from mouse genome (Figure 1A). Analysis of the size distribution and abundance of all sequences within libraries between 17–30 nt revealed two major classes peaking at 21–23 nt and 25–28 nt (Figure 1B, C). Accordingly to the sequence analysis and genomic mapping, the first class of reads with a predominance of 22 nt size was ascribed to miRNAs (Figure 1B, D). 36.71% and 25.21% of mapped reads were assigned to known miRNAs in sugar-fed females and blood-fed females, respectively (Figure 1D). In the second class, 26.02% and 36.00% of mapped reads were associated with repetitive elements in sugar and blood-fed mosquitoes, respectively (Figure 1D). Interestingly, the proportion of mapped reads derived from tRNAs and rRNAs was increased about 1.6-fold in blood-fed compared with sugar-fed mosquitoes (Figure 1D). This likely reflected the beginning of protein synthesis triggered by blood meal intake.

Using miRDeep2 pipeline, we provided transcriptional evidence based on the 5p- and 3p-associated read abundance for 65 distinct *Anopheles* miRNAs previously reported in miRBase (Additional file 1: Figures S1-2). We identified bantam, miR-263a, miR-8, miR-10, miR-184 and miR-281 as the most abundantly expressed miRNAs in *A. gambiae* (Additional file 1: Figure S2). Among these miRNAs, only miR-184 has been previously characterized as the most frequently occurring miRNA in other mosquito species, *Ae. aegypti, Cx. quinquefasciatus* and *Ae. albopictus* [30, 31]. Interestingly, a low number of reads supporting the 3p-associated sequence and absence of the 5p-associated reads were detected for the originally annotated by sequence similarity, *mir-309* and *mir-286* (Additional file 2: Table S1). The extremely low abundance of these miRNAs is probably due to their restricted spatial and temporal expression. Indeed, in *Ae. aegypti* orthologs of miR-309 and miR-286 were detected in embryos and not in the adult females [30]. Overall, these data support the authenticity of the originally annotated set of *A. gambiae* miRNAs in miRBase.

### Identification of novel *A. gambiae*miRNAs

To detect putative *A. gambiae* miRNAs, we used miRDeep2 and a pipeline developed in-house. Only mapped sequence reads were used for the further prediction and expression analyses. We considered a general guideline for microRNA annotation in deep-sequencing data [32–34]. To refine results of the novel miRNA prediction, the following criteria were applied: i) detection of at least 10 sequence reads mapping to the miRNA hairpin per library; ii) valid miRNA precursors folding into stem-loop hairpins with folding energy less than −15 kcal/mol; iii) consistency of the 5’-end starting position, measured as an abundance of mapped sequence reads sharing the same 5’-terminus. Genuine miRNA loci produce so-called mature (miR) and star (miR*) sequences (originated from the preferred and non-preferred strands of miRNA duplex, respectively) that can be derived from both arms of the miRNA precursor. Hence, detection of miRNA sequence reads associated with the 5’- and 3’-arms of miRNA precursor increases a confidence in the novel miRNA annotation [32, 33, 35]. However, it has been reported that certain miRNA might lack miR* reads due to strong asymmetric strand selection during miRNA processing [34]. Therefore, in our study an absence of sequence reads supporting miR* was not considered as a decisive criterion for the annotation of low abundant novel miRNAs. To improve the accuracy of prediction of the novel miRNAs, only miRNAs identified at least in two independent cDNA libraries and in two biological experiments were included in the further analyses. Furthermore, potential novel miRNAs mapped to unknown contigs not associated with any of the three *A. gambaie* chromosomes, with multiple and inexactly mapped reads were filtered out.

In total, 123 distinct miRNAs were detected in the mosquito small RNA libraries (Additional file 2: Table S1), including distinct 65 known and 58 putative novel *Anopheles* miRNAs (Additional file 1: Figure S1 and Table 1, respectively). Sequence alignment revealed that 19 novel miRNAs shared a high level of sequence similarity with the miRNAs described in other insect species. Interestingly, although the *Anopheles* homolog of *mir-71* was not predicted by miRDeep2 in the original data set, the pipeline developed in-house revealed the corresponding 5p- and 3p-associated sequence reads in our libraries (Table 1).

**Table 1:** **Novel** ***A. gambiae*** **miRNAs**

miR sequence	Seed	Predominant arm usage	5p-raw read number	3p-raw read number	Conservation
**GUCGACAGAGAGAUAAAUCACU**	**UCGACAG**	**3p**	**5649**	**207160**	**aae-miR-2940**
**UGUUAACUGUAAGACUGUGUCU**	**GUUAACU**	**3p**	**17**	**76143**	**aae-miR-999**
**CUAAGUACUAGUGCCGCAGGAG**	**UAAGUAC**	**5p**	**63196**	**396**	**aae-miR-252**
**UAGCACCAUUCGAAAUCAGUAC**	**AGCACCA**	**3p**	**63**	**8597**	**aae-miR-285**
**UCAAUUCCGUAGUGCAUUGCAGU**	**CAAUUCC**	**5p**	**3981**	**39**	**aae-miR-932**
**GUAGGCCGGCGGAAACUACUUGC**	**UAGGCCG**	**3p**	**10**	**2313**	**bmo-miR-2796**
**UGACUAGAGGCAGACUCGUUUG**	**GACUAGA**	**3p**	**195**	**1637**	**aae-miR-2945**
**UAGCACCAUGAGAUUCAGCUC**	**AGCACCA**	**3p**	**86**	**1674**	**aae-miR-998**
**UGACUAGACCGAACACUCGUAUC**	**GACUAGA**	**3p**	**4**	**503**	**aae-miR-286b**
**UUGGUGUUAUAUCUUACAGUGAG**	**UGGUGUU**	**3p**	**1**	**597**	**dme-miR-971**
**GAAGGAACUUCUGCUGUGAUCU**	**AAGGAAC**	**5p**	**142**	**7**	**aae-miR-2944a**
**GAAGGAACUCCCGGUGUGAUAUG**	**AAGGAAC**	**5p**	**104**	**21**	**aae-miR-2944b**
**UAUCACAGCCAGCUUUGAAGA**	**AUCACAG**	**3p**	**1858**	**25519**	**aae-miR-2a**
**UGGCAAGAUGUUGGCAUAGCU**	**GGCAAGA**	**5p**	**24655**	**180**	**aae-miR-31**
**AUUAGAAUGUGGAAUCUGUUUUU**	**UUAGAAU**	**5p**	**1572**	**803**	**hsa-miR-561 seed, conserved in aae, cqu**
**UGAACACCCAUUUAUUGCCGACAGG**	**GAACACC**	**3p**	**12**	**1341**	**miR conserved in aae, miR-N3**
**CCCUGUGGAACACCAUGUACGAUGG**	**CCUGUGG**	**3p**	**0**	**202**	**bmo-miR-3389 seed, conserved in aae**
**CAGUACUUCUGCAAUGCAACCC**	**AGUACUU**	**3p**	**113**	**1577**	**aae-miR-33**
**AUGGAUUCGAUCGAUCGAGUGC**	**UGGAUUC**	**5**	**236**	**48**	**dme-miR-976 seed**
**UAGUACGAAUACGUACGAGGGA**	**AGUACGA**	**3p**	**17**	**61**	**aae-miR-2946 seed**
**UGUGGUGGCACACUUUGACAAC**	**GUGGUGG**	**3p**	**2**	**15**	**tca-miR-3897 seed**
**AAUGGCACUCUUGUUGGACAAG**	**AUGGCAC**	**5p**	**24**	**2**	**aae-miR-263a seed**
*CGAUACACGAACUGGGGCUCUCUCC	GAUACAC	3p	49	139	dme-miR-318 seed
**UUAUACUUCCUGCUUCACCGAU**	**UAUACUU**	3p	**0**	**120**	**aae-miR-305 seed**
**GAUUUGUCCAAAAAGGAUG**	**AUUUGUC**	**3p**	**0**	**14**	**aae-miR-981**
**AGGAUUACGAUGAAGUGUUUGCGCC**	**GGAUUAC**	**5p**	**135**	**0**	**bmo-miR-2846 seed, conserved in aae**
**AGAAAGACAUGGGUAGUGAGAU**	**GAAAGAC**	**3p**	**1512**	**13384**	**aae-mir-71**
**ACACGAUAAGAGGAAAGUUUACG**	**CACGAUA**	**5p**	**79**	**11**	**No, miR-N1a (Figure** 5 **D)**
*UUGAAUUACGUCGGCAAUUUUUGGG	UGAAUUA	3p	2	518	hsa-miR-183 seed, miR-N4
**UUAGAUUCCCAGAUCGUCAGAU**	**UAGAUUC**	**3p**	**1**	**48**	**hsa-miR-376a seed**
**UAAGUGCAAAUCGUUGUAGUCGGUU**	**AAGUGCA**	**5p**	**449**	**0**	**hsa-miR-519b seed, miR-N6**
**UUGACUGUCGCCUCUGCGGAUG**	**UGACUGU**	**5p**	**103**	**0**	**hsa-miR-943 seed**
**UUGGAGAUCAAAAGACGAUGUUUUU**	**UGGAGAU**	**5p**	**20**	**2**	**hsa-miR-1270 seed**
**AUGGGGUUUGACCUGCUGGGC**	**UGGGGUU**	**5p**	**41**	**0**	**hsa-miR-3170 seed**
*UCUCCGUGGACGGCUGUCGAUGCC	CUCCGUG	5p	148	1	hsa-miR-3605 seed
**CGGGCUGUUGCAGCAGGUGCCU**	**GGGCUGU**	**3p**	**0**	**67**	**hsa-miR-4741 seed**
**GUAGUCCGGAGUGGAGUC**	**UAGUCCG**	**5p**	**30**	**0**	**hsa-miR-6723 seed**
*UAGGCCCGACCAGAACUCGCUG	AGGCCCG	3p	0	12	hsa-miR-4747 seed
**AACGAGUUUCCCGAUACGACUG**	**ACGAGUU**	**5p**	**282**	**4**	**No**
**CAUUACCGAUGGAUCCUUACCG**	**AUUACCG**	**5p**	**187**	**28**	**No, miR-N2 (Figure** 5 **D)**
**UGCAUUCAGUGGGGCGGUCGU**	**GCAUUCA**	**3p**	**1**	**103**	**No**
**UAGACGAUUUCGGAAUGGCACAUCC**	**AGACGAU**	**3p**	**5**	**492**	**No**
**CCGGUGAACUGCUGUGCAGGGGCGC**	**CGGUGAA**	**3p**	**3**	**209**	**No**
**UCCGGCUACCGACUAACGGCUC**	**CCGGCUA**	**3p**	**2**	**68**	**No**
**ACUCCGGUCGACUCUGGACGAC**	**CUCCGGU**	**3p**	**11**	**34**	**No**
**UUGCGAGAGGACCUAUAAUGACU**	**UGCGAGA**	**5p**	**35**	**0**	**No**
**UUUUGGAACACAAGCUCGGCAGGCC**	**UUUGGAA**	**3p**	**0**	**241**	**No**
**AAUUGGACUCUAUAGCACCCU**	**AUUGGAC**	**3p**	**3**	**78**	**No**
**AACCGACAGAUCAUUGGCCAGA**	**ACCGACA**	**3p**	**0**	**2243**	**No**
**AGGAUUCGUAGUGCUACUGUGCAGA**	**GGAUUCG**	**5p**	**249**	**1**	**No**
**UACUUUCGCAAAUAGAUCGCUGCCU**	**ACUUUCG**	**5p**	**617**	**2**	**No, miR-N5**
**UUGGUCUGAUUGCCUACACUGGCUU**	**UGGUCUG**	**5p**	**497**	**4**	**No**
**UAGGAUCUAUUGACAUUGCAGCCU**	**AGGAUCU**	**5p**	**145**	**0**	**No**
**CUCGCUGGCUGUCCGCAAACU**	**UCGCUGG**	**3p**	**12**	**62**	**No**
**UGAGAGAACGAAAGCAUUCCUU**	**GAGAGAA**	**3p**	**1**	**34**	**No**
**AAUUGGACUCUGUGGCACCCU**	**AUUGGAC**	**5p**	**62**	**0**	**No**
**CGCUCGACUAUUUAUCGCCCGAGA**	**GCUCGAC**	**3p**	**2**	**252**	**No**
*ACGAGGCGAAGACUUUGUUGCC	CGAGGCG	3p	2	12	No

*low confidence candidate miRNA.

**aae -** *Ae. aegypti.*

**cqu -** *C. quinquefasciatus.*

**dme -** *D. melanogaster.*

**bmo -** *B. mori.*

**hsa -** *H. sapiens.*

**tca -** *T. castaneum.*

Evolutionary conservation of miRNA hairpin is considered as a reliable criterion for prediction of novel miRNA genes. Remarkably however, the degree of miRNA conservation does not always correlate with sequence read abundance [35]. For example, we observed that the non-preferred strand of novel *Anopheles mir-981* and *mir-33* hairpins exhibit a high level of sequence similarity (80% and 100%, respectively) with known homologous hairpins. In contrast, the most abundant preferred strand of *mir-33* exhibited sequence divergence in the seed region. Moreover, the preferred strand of *mir-981* shows no significant sequence similarity with any known miRNAs. Since the preferred strand selection of certain miRNAs is not consistently associated with sequence conservation, our data demonstrate the limitations of miRNA prediction and annotation based on sequence similarity. We also detected 17 miRNAs with a sequence similarity restricted to the seed sequence only. Among those, putative miRNA orthologs were found for 4 *Anopheles* miRNAs in the closely related mosquito species *Ae. aegypti* and *Cx. quinquefasciatus* (Table 1; Additional file 1: Figure S3). Finally, the remaining 21 *Anopheles* miRNAs had no obvious sequence similarity with known miRNAs in other species (Table 1).

Closer examination of mature sequences derived from both arms of the predicted hairpins revealed that 20 novel candidate miRNA loci were associated with the presence of distal and proximal sequence reads. This might indicate that the candidate miRNA is a degradation intermediate [32]. Recently however, an example of a miRNA locus showing signatures of both, host mRNA degradation and miRNA processing via Drosha/Dicer cleavage has been reported in *Drosophila* [34]. 80% of those *Anopheles* miRNAs mapped to intergenic regions. Yet, the possibility of dubious annotation of the predicted protein-coding genes and intergenic regions in Vector Base cannot be excluded. Manual inspection of the miR/miR* sequence reads comprising putative miRNA duplexes revealed that 70% of these miRNAs exhibit expected 1–2 nt 3’-end overhangs, the signature of substrates processed by the RNAse III enzymes, Drosha/Dicer. The remaining miRNA loci, including intron-derived (mirtrons) and intergenic miRNAs show unusual 3’-end overhangs. Among those, three intergenic-derived putative miRNAs (*dme-mir-318*, *hsa-mir-3605* and *hsa-mir-183* seeds) exhibit very atypical 3’-end overhangs. Those miRNAs were considered to be low-confidence candidate miRNAs (Table 1); and therefore further experimental studies will be required to demonstrate their functional association with miRISC.

### Validation of novel miRNA candidates

**Figure 2:**
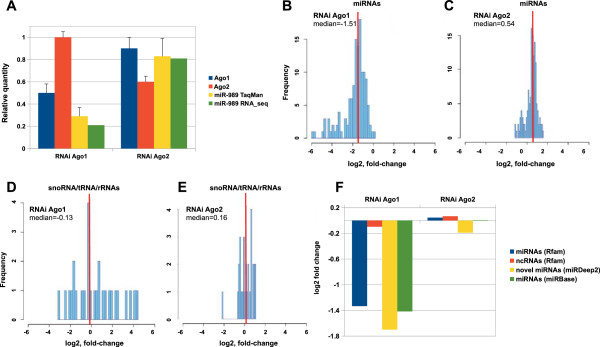
**Effect of** ***Ago1*** **and** ***Ago2*** **silencing on small RNA levels. (A)** Relative expression levels of *Ago1-2* and miR-989 in small RNA libraries after RNA silencing. dsRNA injection was used to deplete *Ago1* and *Ago2* in adult females. Relative quantity of mature miR-989 was measured by TaqMan assay and compared with miR-989 sequence read number in *Ago1-* and *Ago2*-silenced mosquitoes. Fold-changes in small RNA levels in *Ago1-* and *Ago2*-silenced mosquitoes **(B, C)** for miRNAs (miRBase and miRDeep2), **(D, E)** tRNA/rRNA/snoRNAs (Rfam); the median fold-change is shown for each plot. **(F)** Fold-changes in miRNA expression levels (Rfam, miRDeep2 and miRBase) normalized by snRNA U2 in *Ago*-silenced mosquitoes as indicated*.*

Mature miRNA levels and stability require predominantly AGO effector proteins. Therefore, to increase the specificity of novel miRNA prediction, we analyzed miRNA levels in *Ago1*-silenced mosquitoes. As a control, we used mosquitoes depleted for AGO2, the effector of siRNAs. Small RNA libraries were prepared from mosquitoes injected with dsRNA against *Ago1* and *Ago2* and sequenced on the Illumina platform. Quantification of relative expression by qPCR showed that in the *Ago1*- and *Ago2-*silenced mosquitoes levels of *Ago1* and *Ago2* were downregulated by 50% and 40%, respectively (Figure 2A). Importantly, no cross-silencing of *Ago1* and *Ago2* expression was detected suggesting that the observed silencing effect was specific to the corresponding target mRNA (Figure 2A). The TaqMan-based quantification and validation of the previously annotated miR-989 showed that the miR-989 expression levels were decreased by 65% in *Ago1*-silenced mosquitoes (Figure 2A). Accordingly to normalized RNA sequence read quantification in *Ago*-silenced libraries, miR-989 levels were decreased by ~80% in *Ago1*-silenced mosquitoes, while in *Ago2*-silenced mosquitoes miR-989 levels have not been substantially changed (Figure 2A). The relative quantification of mature miR-989 in our small RNA libraries was consistent with qPCR measurements. We then investigated the effect of *Ago1* and *Ago2* silencing on the total miRNA expression levels in the dsRNA injected mosquitoes, using non-injected mosquitoes as a reference control (Figure 2B, C). We observed a 65% decrease in the total miRNA levels in *Ago1*-silenced mosquitoes, corresponding to a median log_2_ fold-change of −1.51 (Figure 2B). Silencing of *Ago2* resulted in a log_2_ fold-change of 0.54 in miRNA expression levels (Figure 2C). In addition, as an internal negative control, we analyzed levels of small RNAs unrelated to the miRNA pathways. For snoRNAs, tRNAs and rRNAs, we detected 9% decrease in *Ago1*-silenced mosquitoes (Figure 2D), that corresponds to the median log_2_ fold-change of −0.13, while in *Ago2*-silenced mosquitoes the median log_2_ fold-change of snoRNAs, tRNAs and rRNAs levels was 0.16 (Figure 2E).

We next examined individual changes in the expression levels of the newly identified miRNAs in *Ago1-* and *Ago2*-silenced libraries using expression of known *Anopheles* miRNAs as a positive control (Figure 2F). A small non-coding RNA not related to miRNAs U2 snRNA, invariantly expressed in all our libraries, was used to normalize miRNA expression levels. Similar log_2_ fold-changes (−1.6) were observed for the expression levels of the newly identified miRNAs (miRDeep2) in *Ago1*-silenced mosquitoes as compared to the known miRNAs annotated in miRBase (−1.4) and Rfam (−1.3). In *Ago2*-silenced control, we did not observe substantial changes in the analyzed miRNA expression levels (Figure 2F). Furthermore, the expression levels of ncRNAs, unrelated to miRNAs (Rfam) were consistent in both *Ago1-* and *Ago2-*silenced libraries (Figure 2F). Remarkably, only one newly predicted miRNA (*hsa-mir-4747* seed) with an extremely low number of reads showed no changes in expression levels in *Ago1*-silenced mosquitoes, therefore, it was not considered as a confident miRNA candidate. Collectively, these data suggested that levels of mature *Anopheles* miRNAs were specifically affected by *Ago1* silencing, thereby supporting the accuracy of the miRNA prediction and annotation in this study.

### miRNA sequence heterogeneity

Deep sequencing analyses revealed sequence heterogeneity at the 5’- and 3‘-end of mature miRNA sequences, collectively called iso-miR variations. Such sequence variations can occur due to inaccurate processing by Drosha/Dicer-1, degradation and non-template sequence extension [19, 20, 34, 36–41]. Analysis of sequence variations due to mismatches between the reads in our libraries and their corresponding genomic loci revealed ~6.7% reads within mature miRNA sequences with 1 nt and more mismatching from their genomic loci. It has been reported that miRNA might be subjected to RNA editing by adenosine deaminase (A to G transition) and cytidine deaminase (C to U transition) [34, 38]. Therefore, we analyzed occurrences of the putatively edited mature miRNA sequence reads in our libraries. We did not find any evidence for the enrichment of A to G and C to U changes compared with other types of nucleotide alterations. Therefore, the observed sequence variations detected within mature miRNAs can be attributed to sequencing errors and/or to sequence variations between the query and the reference genome.

**Figure 3:**
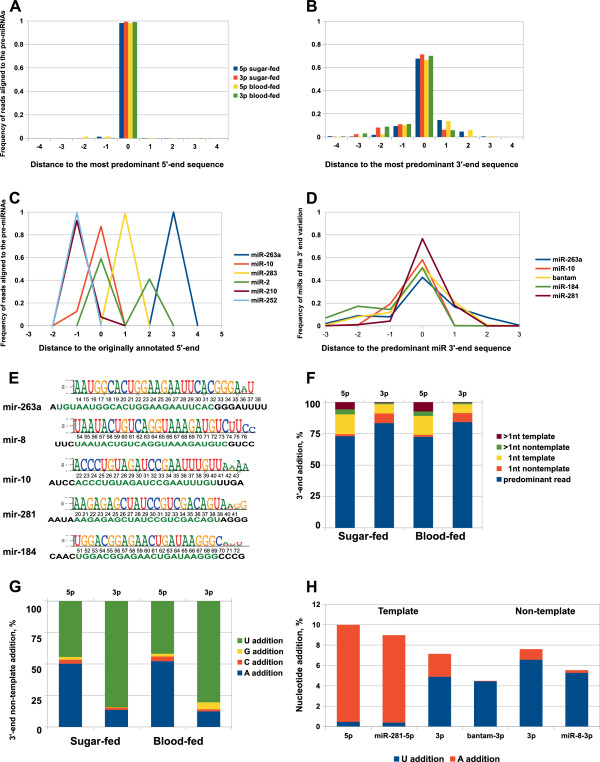
**miRNA sequence variation. (A-B)** 5’- and 3’-end sequence heterogeneity of the 5p- and 3p-derived reads as indicated. **(C)** Distribution of the predominant sequence reads grouped by their 5’-ends compared with the miRNAs reported in miRBase v19. **(D)** Frequency of sequence reads grouped by their 3’-end compared to the predominant miRNA read. **(E)** Sequence compilations of five highly abundant mosquito miRNAs showing their mature sequence including the corresponding 3’-end sequence variations. Below, the mature miRNA sequence reported in miRBase is shown in green, adjacent genomic sequence is in black. **(F-G)** 3‘-end extensions of the 5p- and 3p-sequences in sugar and blood-fed mosquitoes as indicated. **(H)** The percentage of A- and U-tailed sequence reads of the extremely abundant miRNAs.

The specificity of target recognition is mostly determined by the 5’-end of miRNAs [12]. Furthermore, the 5’-end precision and homogeneity of mature miRNAs show a high degree of evolutionary constraint. Therefore, we carefully analyzed cleavage accuracy of miRNA sequence reads associated with the 5’- and 3’-arms of miRNA precursors in our libraries. We detected high 5’-end fidelity of the 5p- and 3p-associated sequence reads that was nearly identical (Figure 3A). Similar tendency was observed for the 5’-end homogeneity of mature and star miRNA sequences (Additional file 1: Figure S4). Importantly, we identified a group of miRNAs, including miR-283, miR-2, miR-210, miR-263a, miR-10 and miR-252, with heterogenous 5’-ends (Figure 3C). *mir-2-1* and *mir-2-2* have been previously reported in miRBase as a miRNA with identical mature sequence, referred to as miR-2b and miR-2c, respectively. For miR-2, there were two abundant classes of the 5’-end, the one with the originally reported in miRBase and another, which was with 2 nt shorter (Figure 3C). In *Drosophila*, *mir-2* subjected to alternative processing also produces two distinct miR-2 isoforms with 2 nt shifted 5’-ends with respect to each other [42]. We identified miR-10 and miR-210 with an extra 5’ cytosine matched to the sequence of the pre-miRNA. For miR-10, two dominant mature species occur with the originally annotated 5’-end (~87% of reads) and with an extra 5’ cytosine (~12% of reads) (Figure 3C). Similar variation at the 5’-end has been described in *D. melanogaster* miR-10 and miR-210, illustrating an example of the single hairpin generating mature miR with different abundant 5’-ends [34, 37]. In *Cx. quinquefasciatus*, two dominant iso-miR species have been reported for miR-210, one of which contains an additonal cytosine nucleotide at its 5’-end [31]. Interestingly, *A. gambiae* miR-210 has only one dominant species with 5’-end cytosine addition (~92% of reads). The originally annotated 5’-end for miR-210 (miRBase) is represented by a small fraction of reads (7.6%) in our libraries. Since in *A. gambiae* there are no paralogs of miR-10 and miR-210, this difference cannot be due to processing of mature miR from distinct homologous precursors. In *Cx. quinquefasciatus*, two dominant species have been reported for miR-252, one of which is 1 nt longer, with an extra cytosine residue at the 5’-end [31]. Similar modification at their 5’-end were detected in *A. albopictus* miR-252 (35% of mature reads) [31]. For miR-252 identified in this study, we found 99% of mature reads with a template-directed cytosine addition at the 5’-end (Figure 3C). This consistent 5’-end cytosine addition had also been reported for *Aedes* miR-252 [30]. Interestingly, the predominant sequence of miR-263a was associated with a 3-nt shifted 5’-end; no sequence reads corresponding to the originally annotated sequence (miRBase v19) were detected in our libraries (Figure 3C, E). For miR-283, fewer than 1% of sequence reads corresponded to the mature sequence reported in miRBase v19. Instead, the majority of the annotated reads (more than 98%) were 1 nt shorter at their 5’-ends (Figure 3C). Collectively, the detected variations at the 5’-end of mature miRNAs that result in the functional-seed shifting, were observed for more than 5% of *Anopheles* miRNAs across analyzed libraries.

Significant sequence heterogeneity was also observed at the 3’-end of miRNA sequence reads (Figure 3B cf. 3A; Additional file 1: Figure S4). This is consistent with the general observation that 3’-end sequence variation is more common than 5’-end variation [20, 32, 34, 39]. Within all mature miRNA reads, around 14% reads have undergone more than 1 nt trimming or degradation at the 3’-end. Among the extremely abundant miRNAs, 1–2 nt trimming at the 3’-end was observed for 20% of reads from bantam and miR-10; for 17% of reads from miR-263a and for 32% of reads from miR-184 (Figure 3D). Next we extensively analyzed the 3‘-end sequence extension (tailing) due to template and non-template directed nucleotide additions (Figure 3F, G). Analysis of the 3’-end composition revealed that the 5p-associated sequence reads were preferentially subjected to template-directed extension of one or more nucleotides (Figure 3F). Furthermore, the frequency of non-template-directed additions was substantially lower at the 3’-end of 5p-associated sequences with respect to the 3p-associated reads (Figure 3F). Among the abundant *Anopheles* miRNAs, more than 60% of miR-263a reads were 1 – 3 nt longer at the 3’-end than its mature sequence reported in miRBase (Figure 3D, E), which was represented by ~2% of reads. Moreover, about one quarter of bantam and miR-281 reads was 1 nt longer than their corresponding mature sequences reported in miRBase (Figure 3D, E). The described above nucleotide additions matched to the corresponding pre-miRNA sequences, indicating a template-directed origin of these additions most likely due to imprecise processing. In addition, we observed that miRNA loci, such as *mir-277*, *let-7*, *mir-1174* and *mir-279* produced from one fourth to one third of sequence reads tailed by template-directed adenine addition. Remarkably, a significant proportion of detected template-directed adenine and uracil additions in sequence reads derived from the 5’-arm is associated with miR-281 (Figure 3H). Moreover, the majority of template-directed uracil extensions in the 3’-arm-derived reads are associated with bantam (Figure 3H). It has been reported that sequence variation occurring due to imprecise cleavage by Drosha and Dicer are more frequent than non-template addition [39]. A similar tendency was observed for the 5p-associated sequence variations of *Anopheles* miRNAs (Figure 3F). However, the frequencies of template and non-template-directed additions were near similar at the 3’-end of the 3p-associated sequence reads (Figure 3F). The most predominant non-template directed nucleotide additions associated with the 5p-reads were adenine (44%) and uracil (50%) nucleotides (Figure 3G). We found that miR-125, miR-283, miR-10, miR-100, miR-281* and certain abundant isoforms of miR-279, miR-1174, miR-263a and miR-281 were substantially adenylated. Furthermore, we observed a bias toward uridylation in miRNA sequence reads (around 81-84%) derived from the 3’-arm of the precursor miRNA across analyzed libraries (Figure 3G). Such modifications were mostly associated with miR-11, miR-14, miR-317, miR-277, miR-184, miR-8, miR-92b and miR-989. Strikingly, more than 80% of detected non-template directed uracil additions were associated with miR-8 (Figure 3G, E). The additions of cytosine and guanosine were detected only for 1-5% of sequence reads (Figure 3G). Taken together, the levels of the 3’ uridylation and adenylation were substantially greater than other types of nucleotide additions in *Anopheles* miRNAs. This is consistent with the earlier reported observations for mammalian and insect miRNAs [19, 34].

### Functional arm usage and shifts in sugar and blood-fed mosquitoes

**Figure 4:**
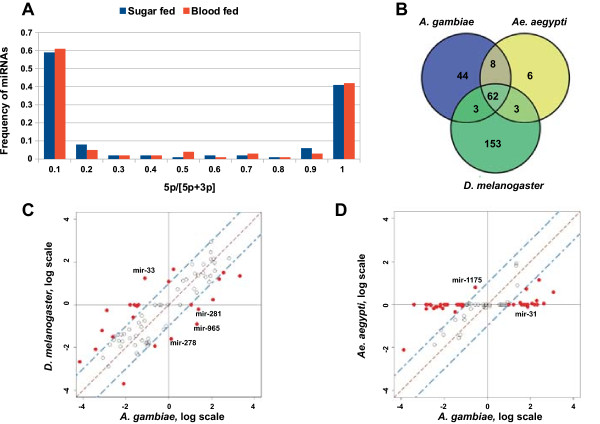
**Arm usage analyses in** ***Anopheles*** **miRNAs. (A)** Proportion of sequence reads associated with the 5’ arm of miRNAs with respect to the total number of reads in sugar and blood-fed *A. gambiae* mosquitoes. **(B)** Venn diagram showing the number of putative *A. gambiae* miRNA homologs in *D. melanogaster* and *Ae. aegypti.* **(C-D)** Comparison of relative arm usage between *D. melanogaster* and *A. gambiae* **(C)** and between two mosquito species, *A. gambiae* and *Ae. aegypti* **(D)**. Shown are miRs exhibiting an arm usage bias; the dashed line indicates 10-fold differences in the relative arm usage.

Analysis of the 5’- and 3’-arm usage in *Drosophila* revealed a slight bias towards 5’-arm usage (35). In contrast, in *A. gambiae,* we observed a bias towards 3’-arm usage (average 5p/3p proportion was 0.4), which was consistent with abundance of sequence reads associated with the 3’-arm of precursor miRNAs (Figure 4A). It has been reported that selection and usage of the preferred arm can be dynamically regulated during development in a tissue-specific manner [20, 32]. Switching in the functional arm usage changes the mature miRNA sequence selection and production, which consequently influences the target repertoire and function of a given miRNA. Notably, sequences associated with the non-preferred arm might also exhibit specific function *in vivo* [43]. To explore the preferred arm selection in *Anopheles* miRNAs, we reanalyzed arm usage reported in miRBase v19 and compared it with our data sets. We identified a group of miRNAs, *mir-133*, *mir-1891*, *mir-278*, *mir-281*, *mir-965*, *mir-929* that produced corresponding miR* at significantly higher levels compared to their mature miRs reported in miRBase (Table 2). Among these miRNAs, the mature miRNA sequence homologous to *Anopheles* miR-281* has been reported earlier as predominantly expressed in *Cx. quinquefasciatus* [31]. In addition, the newly identified *A. gambiae* ortholog of *mir-2940* also predominantly produced miR-2940* across all analyzed libraries (Table 1). Furthermore, we identified two *Anopheles* miRNAs, *mir-305* and *mir-1889* that were not subjected to strong asymmetric strand selection (Table 2). They produced sequence reads associated with the 5’- and 3’-arm of the precursor at nearly the same levels, suggesting that preferred and non-preferred strands of the miRNA duplex are equally processed. The equal strand selection for *mir-1889* has been previously reported in *Ae. albopictus* [31]. Interestingly, a shift from the predominant arm usage to a nearly equal 5p/3p species production was observed for *mir-219* and *mir-3840* in the libraries derived from blood-fed mosquitoes (Table 2). However, overall blood meal intake did not affect the frequency of the relative arm usage more than 10-fold in *A. gambiae* libraries (not shown).

**Table 2:** **Functional arm shifting in** ***A. gambiae*** **miRNAs**

miRNA	miRBase annotated predominant arm	RNA_seq		
		detected predominant arm	5p/3p_SF	5p/3p_BF
miR-133	5p	3p	0.01	0.01
miR-1891	5p	3p	0.37	0.27
miR-278	3p	5p	1.33	1.34
miR-281	3p	5p	24.81	22.59
miR-965	3p	5p	23.56	9.56
miR-929	3p	5p	27.92	14.67
miR-1889	3p	5p~3p	0.93	0.72
miR-305	5p	5p~3p	1.02	0.85
miR-219	5p	5p	1.91	0.91
miR-3840	novel miRNA	3p	0.24	1

SF-sugar-fed.

BF-blood fed.

We next analyzed species-specific arm selection by comparing the relative arm usage between *A. gambiae*, *Ae. aegypti* and *D. melanogaster* (miRNA data sets described in Methods). We retrieved insect miRNA homologs by considering 1:1 orthologous miRNA pairs between these species for the analysis (Figure 4B). Comparison of the relative arm usage revealed four miRNAs switching their preferred arms (Figure 4C). *mir-965*, *mir-281* and *mir-278* predominantly used the 5’-arm in *Anopheles*, whereas the 3’-arm was preferentially used in *Drosophila*. The opposite tendency was observed for *mir-33*. Comparison between sugar-fed *Anopheles* and *Aedes* female mosquitoes revealed no difference in the relative arm usage (not shown). Strikingly, 10-fold greater difference in relative arm usage was observed for *mir-1175* and *mir-31* orthologs in blood-fed *Anopheles* compared to *Aedes* females (Figure 4D). Taken together, our data revealed an existence of species-specific production of dominant mature miRNAs that might be involved in the regulation of the blood meal-induced physiological traits in the mosquito species and could be associated with the species diversification during *Diptera* evolution.

### Genomic organization, duplication and clustering of *Anopheles*miRNA genes

Analysis of genomic organization revealed that around 68% of *A. gambiae* miRNAs were intergenic miRNAs. The remaining 32% showed an overlap with the predicted transcripts annotated in Vector Base often mapping to the coding (sense) strand. Among those, 25% of miRNAs were located in introns, whereas ~7% were mapped to exons. Expression of sense strand-derived miRNAs most likely coincides with expression of the host gene. Only three newly identified miRNAs were on the non-coding (antisense) strands of the overlapping transcripts. Such genomic organization of host genes and antisense miRNAs might have a regulatory function to interfere with miRNA transcription, or might affect the host gene to influence mRNA splicing or to target sense mRNA. Interestingly, known insect-specific antisense transcribed miRNAs *mir-307* and *iab-4* do not exhibit conserved genomic organization in *A. gambiae*. Furthermore, we did not find any examples of miRNAs convergently transcribed from both sense and antisense strands in *A. gambiae*.

**Figure 5:**
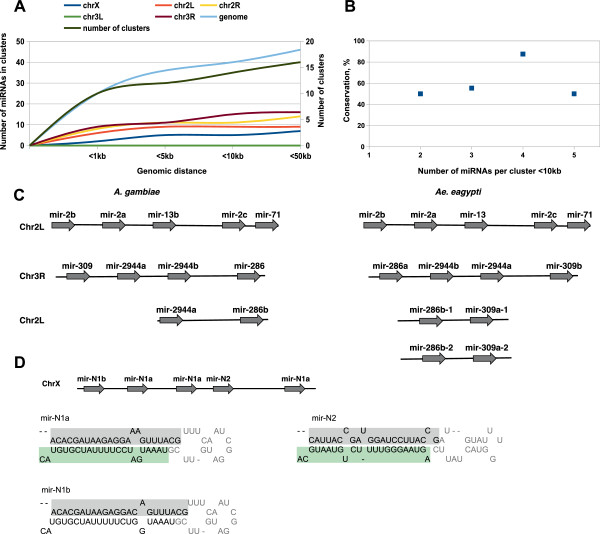
**miRNA cluster analysis in** ***A. gambiae*** **. (A)** Number of miRNAs in clusters in respect to genomic distance in *A. gambiae.* **(B)** Number of miRNAs conserved in clusters between *A. gambiae* and *Ae. aegypti.* **(C)** Conservation of *mir-2/mir-13/mir-71* and *mir-2944/mir-309/mir-286* in *A. gambiae* and *Ae. aegypti.* **(D)** Novel *A. gambiae-specific* clustering miRNA loci that generate miR (grey) and miR* (green) associated sequence reads in mosquito libraries; the predicted pre-miRNA stem-loop structures are shown below.

miRNA gene duplication is an important source of phenotypic plasticity, robustness and diversity in development. The previously described sets of *Anopheles* miRNA paralogous genes include *mir-375*, *mir-965*, *mir-2*, *mir-92*, *mir-9* and *mir-263* (miRBase v19). We analyzed multiple mapped *A. gambiae* miRNAs, scoring the new paralogous miRNAs as a duplication of pre-miRNA precursors and/or mature and star sequences. miRNAs detected in unknown contigs not associated with any of three *A. gambaie* chromosomes, with mismatches to the reference genome were filtered out. We further extended paralogous miRNA gene sets by describing new homologs of known *Anopheles* miRNAs and of new miRNAs annotated in this study (Table 1; Additional file 1: Figures S3; S5). Novel *mir-276* (*mir-276-2)* shares 100% similarity with the mature and star sequences of the originally annotated *mir-276* (miRBase), showing sequence divergence only within the terminal loop. Mature miR-309 sequence was represented by two perfect copies, whereas star and terminal loop sequences of the two *mir-309* paralogous genes were not conserved. Moreover, *mir-2944* identified in this study was represented by three homologous genes: *mir-2944a-1/-2* and *mir-2944b* (Table 1). Finally, we annotated a novel member of the *mir-2* gene family (*mir-2b + c), mir-2a* (Table 1, Figure 5C).

miRNAs are frequently clustered in the genome (miRBase) and are most likely expressed from polycistronic transcripts. We analyzed the organization of miRNA clusters and their evolutionary conservation within the mosquito species (Figure 5A). The analysis of miRNA clusters in *A. gambiae* genome revealed that approximately 40% of miRNAs are clustered on the X chromosome. Around 36% of miRNAs were closely linked on the chromosomes 2 and 3R, however no miRNA clusters were found on the chromosome 3 L. We observed that 20% of *Anopheles* miRNAs were closely linked within 1 kb genomic distance. For larger clusters, around 30% of miRNAs were linked within 5–10 kb genomic distance and approximately 40% of miRNAs were closely linked at the distance of 50 kb and less (Figure 5A). The mean number of miRNAs per cluster was two for 1 kb genomic distance and three miRNAs for 5 – 50 kb genomic distance (Figure 5A). Collectively, these data indicates that one third of *Anopheles* miRNAs are closely linked within 50 kb. We also observed that all closely linked *Anopheles* miRNAs are located on the same stand within analyzed clusters. Expression levels of clustered miRNAs were significantly correlated across all mosquito libraries (R^2^ = 0.99). Nevertheless, further regulation at the processing level might provide the eventual “activity” patterns distinct from the expression patterns for the neighboring miRNAs of the same cluster. The proportion of clustered miRNAs producing the dominant miRNA from the same arm was 0.85 within a distance of 50 kb and less, and 0.57 for non-clustered miRNAs scored at a distance more than 50 kb, revealing a strong bias towards the same arm selection for clustered miRNAs with respect to non-clustered miRNAs.

We further analyzed a conservation of clustered miRNAs between *Anopheles* and *Aedes* genomes within 10 kb of genomic distance (Figure 5B). Since the repertoire of miRNAs largely overlaps between these mosquito species, comparison of miRNA clusters consistently revealed a high level of conservation between *A. gambiae* and *Ae. aegypti*. Around 68% of clusters containing four miRNAs maintained their closely linked organization in both species. Moreover, more than 75% of conserved orthologous miRNAs grouped in clusters of two, three and five miRNAs were linked in *Aedes*. Importantly, this analysis allowed us to revise the clustered miRNA sets in *Anopheles*. We further extended previously predicted by sequence similarity *mir-2/mir-13* cluster (miRBase v19) by providing evidences for *mir-2a* and *mir-71* expression in our study (Figure 5C). Notably, the organization of *mir-2/mir-13/mir-71* cluster is highly conserved in invertebrates [35]. Other examples are the newly described *mir-2944a* and *mir-2944b*, which formed a cluster with the previously annotated *mir-309* and *mir-286* (Figure 5C). Interestingly, we identified a fragmented duplication of the *mir-2944/mir-309/mir-286* cluster, which included only *mir-2944a/mir-286* and lacked *mir-309*. In *Ae. aegypti,* the fragmented cluster contains only *mir-286* and *mir-309*, lacking *mir-2944*. Although clustering of *mir-2944/mir-309/mir-286* was conserved between *A. gambiae* and *Ae. aegypti,* the fragmented clusters described above represent an example of miRNA cluster diversification between these mosquito species. *Anopheles mir-285,* identified in this study, is closely linked with *mir-11* at a distance shorter than 1 kb. However, the clustering organization of these miRNAs is not conserved in *Aedes*. In *A. gambiae, mir-965* was represented by a cluster of two paralogous genes, *mir-965-1* and *mir-965-2*, whereas only a single *mir-965* ortholog was described in *Aedes*. Finally, we identified a novel cluster containing two *A. gambiae*-specific *mir-N1* and *mir-N2* that shared no significant homology with any known miRNAs (Figure 5D). The miRNA expression profiling revealed a relatively low abundance of these miRNAs (Table 1). miR-N1 might potentially arise from four clustered hairpin precursors. Three out of four hairpin precursors represented a perfect duplication of *mir-N1*. The fourth hairpin precursor was slightly divergent showing minor sequence changes in the mature and star sequences supported by sequence reads in our libraries. In summary, miRNA clustering is highly conserved between *Aedes* and *Anopheles* indicating the orthologous origin of these clusters. The described *A. gambiae*-specific miRNA gene clustering and cluster fragmentation are most likely an example of the evolutionary “young” species-specific miRNA segregation emerging through gene duplication followed by sequence divergence due to mutational drift.

### Regulation of miRNA expression by regular and infectious blood feeding

**Figure 6:**
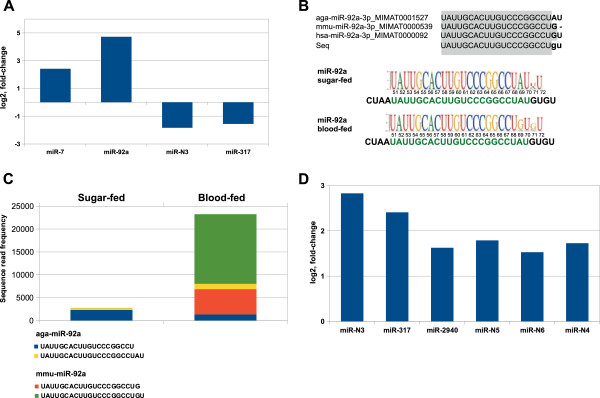
**Differential expression of miRNAs in** ***Anopheles*** **females 3 h after regular and infectious blood feeding. (A)** Fold-changes in miRNA expression are shown as a ratio of blood-fed to sugar-fed mosquitoes (*p > 0.05*). **(B)** Multiple RNA sequence alignment for mouse and human homologs of *Anopheles* miR-92a (miRBase v19). Sequence compilations of miR-92a mature sequence with the 3’-end variation in the mosquito libraries as indicated. **(C)** Sequence read abundance corresponding to two abundant classes of aga-miR-92a and mmu-miR-92a in sugar and blood-fed mosquitoes are as indicated. **(D)** Differentially expressed miRNAs in *P. berghei* infected mosquitoes; the fold-changes in miRNA expression levels are shown as a ratio of infected to non-infected blood-fed mosquito samples (*p > 0.05*).

In order to identify miRNAs regulated by blood feeding, we first compared the relative abundance of miRNAs in sugar-fed mosquitoes and in mosquitoes 3 h after a feeding on a mouse (Figure 4A). Using log_2_ fold-change, more than 1.5 as a threshold, we revealed changes in abundance of miR-7, miR-92a, miR-317 and newly described miR-N3 (Figure 6A). The expression levels of abundant miR-7 and miR-92a were more than 5- and 25-fold upregulated by blood feeding, respectively. miR-92a is a highly conserved miRNA in animals, including the mammalian blood-hosts of mosquitoes, with minor species-specific sequence variations at the 3’ end (Figure 6B). Therefore, we examined the origin of miR-92a elevated levels in the analyzed libraries. The analysis of miR-92a-associated sequence reads in sugar-fed mosquitoes revealed that miR-92a was represented by two classes: a 22 nt-long mature sequence reported in miRBase as aga-miR-92a and another sequence of a 20 nt (Figure 6B, C). In blood-fed females, four distinct predominant miR-92a classes were detected, including the above described *Anopheles*-specific mature sequences and two mature sequences assigned to *M. musculus* (miRBase v19). Around 90% of the total mature miR-92a reads in blood-fed mosquitoes were associated with the two isoforms of mmu-miR-92a (Figure 6B, C). Interestingly, the predominant mouse mmu-miR-92a sequence isoform detected in the blood-fed libraries was identical with the human hsa-miR-92a sequence reported in miRBase (Figure 6B). No significant changes were observed in the levels of endogenous *Anopheles* aga-miR-92a (Figure 6C). Taken together our data suggest that the dramatic change in miR-92a levels resulted from the exogenous miRNA intake occurred during blood feeding.

To assess the early responses of *Anopheles* miRNAs specific to *P. berghei* infection, we compared miRNA expression levels in females 3 h after regular and infectious blood feeding. We identified 6 miRNAs responding to the *P. berghei* parasite presence (Figure 6D). Among those, two highly expressed miRNAs, miR-317 and miR-2940 were more than 5- and 3-fold upregulated by *P. berghei* infection, respectively. The remaining differentially expressed miRNAs showed a relatively low abundance. Overall, the *Anopheles* miRNAs identified here might represent early and dynamically regulated mosquito sensors that respond to normal and infectious blood meals.

## Discussion

In the current study, using small RNA sequencing, we performed the first systematic analysis of *Anopheles* miRNAs and iso-miRs. Highly expressed *Anopheles* miRNAs (e.g. bantam or miR-263a) are represented by numerous mature sequence variants that vary in their abundance across all analyzed libraries. We characterized 5’-end variations in detail, which alter miRNA seed sequences, thereby diversifying the target specificity of a given miRNA. We showed that more than 5% of *Anopheles* miRNAs exhibit shifts in the functional seed sequences. These include newly described predominant isoforms of known *Anopheles* miRNAs, such as miR-263a, miR-283, or miR-210 that are abundantly expressed across all libraries. The detected here predominant mature sequences of these miRNAs are distinct from those originally predicted by sequence similarity (miRBase). Interestingly, *Anopheles* miRNA loci such as *mir-2b/-c* are capable of producing equal numbers of the highly expressed iso-miR species with alternative 5’-ends. Remarkably, *Drosophila* homologous *mir-2* is also subject to alternative Dicer processing [42]. The origin of the conserved processing pattern of *mir-2* homologs is unclear. It has been reported recently, that the alternative length of mature miRNAs can be regulated by the interacting partners of RNAse III enzymes [44]. Therefore, generation of alternative iso-miR-2 may require specific trans-acting factors regulating precision of Drosha/Dicer cleavage.

We also laboriously characterized miRNA sequence heterogeneity at 3’-ends (Figure 3, Additional file 1: Figure S4). The substantial fraction of *Anopheles* miRNAs exhibits 3’-end trimming and additions. Among nongenome-matching (non-template-directed) 3’-nucleotide extensions, uracil and adenine additions were the most predominant modifications of the mature miRNA sequences across analyzed mosquito libraries. Strikingly, in *A. gambiae,* 80% of nontemplate-directed urydilated reads derived from miR-8. The number of described 3’ additions in this study might be significantly underestimated since certain adenine and uracil additions map to adjacent genomic sequence of the pre-miRNA (e.g. miR-281, let-7 or bantam) and therefore, it was not possible to discern unambiguously between template- and nontemplate-directed additions. It has been reported that 3’ adenylation stabilizes miRNAs [40, 45], whereas uridylation targets miRNAs for degradation (21,41). In other studies, a bias towards 3’ uridylation was observed for the AGO-immunoprecipitated miRNA fraction [20]. In contrast, it has been shown that 3’ adenylation correlated with the reduced association between miRNAs and miRISC [19, 20]. Regardless of how this controversy is ultimately resolved, both modifications can impact miRNA processing and activity profoundly.

Functions of the most abundantly tailed miR-8, bantam and miR-281 are not known yet in *A. gambiae*. It has been reported that bantam downregulates ecdysone signaling during larval development in *Drosophila* [46]. In addition, the ecdysone-responsive *Drosophila* miR-8 regulates insulin signaling and innate immune homeostasis [47, 48]. The comparison of consistently adenylated and uridylated miRNA sets identified in this study and reported in [34] revealed that the described 3’-end additions are not conserved in *Drosophila* homologous miRNAs. Nevertheless, the detected 3’ extensions of miR-8 and bantam might be a signature of their activity in the complex regulatory network of insulin and ecdysone signaling in *A. gambiae* females triggered by blood meal intake.

The *Anopheles* genus separated approximately 120–190 milllion years ago from another blood-feeding mosquito genus *Aedes*, which is the main arbovirus vector responsible for transmitting *alpha*- and *flavi*viruses to humans. Despite the divergence and complexity of the *Aedes* genome with respect to *Anopheles*, the comparative analysis of our miRNA data sets and *Ae. aegypti* miRNAs revealed that more than a half of miRNAs were evolutionary conserved between these species. Overall, the relative arm usage of homologous miRNA loci was largely consistent in both *Anopheles* and *Aedes*. Yet, our study revealed an example of a remarkable change in the relative arm usage frequency in *mir-1175* and *mir-31* loci in response to blood feeding. One third of *Anopheles* miRNAs are closely linked in the genome, and such clustering organization is largely conserved between *Aedes* and *Anopheles*. Importantly, we described *de novo* emergence of species-specific miRNAs together with miRNA gene duplication and/or segregation in new clusters and further cluster fragmentation, which might be important for shaping of vector competence traits in these insects.

Functional arm switching significantly diversifies the regulatory capacity of miRNA genes. Selection of the preferred arm is regulated in a tissue- and organ-specific manner during development [20, 32]. We did not detect dramatic changes in arm switching between sugar- and blood-fed *Anopheles* mosquitoes, whereas substantial fluctuations in the 5’/3’-arm usage were observed. Interestingly, we identified two examples of *Anopheles* miRNA loci, *mir-305* and *mir-1889* with absence of strong asymmetry in the preferred strand selection (Table 2). Furthermore, the preferred arm usage in *mir-219* and *mir-3840* loci was tilted after blood feeding to a nearly equal production of mature and star sequence species. All together, our extended set of *Anopheles* miRNAs and their isoforms provides a ground for further experimental studies of miRNA patterns and biological functions in *A. gambiae*.

Comparative analysis of miRNA abundance revealed dramatically elevated levels of miR-92a after blood meal intake. Interestingly, the sequence fraction assigned to miR-92a was enriched in mmu-miR-92a derived from the blood-host mouse. The mammalian miR-92a is a member of the conserved *mir-17-92* cluster, whose over-expression is associated with lymphomas and other cancers [49]. Characterization of miRNA expression profiles in human blood revealed abundant expression of miR-92 in mature erythrocytes [50]. Consistent with this observation, elevated levels of human miR-92a were detected in the mosquito small RNA libraries prepared from females fed on human donor blood (not shown). We identified a set of highly abundant murine miRNAs in mosquito females fed on regular and infected blood (Additional file 3: Table S2). Interestingly, the *P. berghei* infection was associated with significant changes in abundance of *mmu-mir-5105*, *mmu-mir-5115*, *mmu-mir-6243* and *mmu-mir-5109*. It has been reported that host blood-derived factors, such as human insulin, can modulate immunity and susceptibility of *Anopheles* mosquitoes to human *Plasmodium* infections [1]. However, the function of miRNAs derived from host-blood in mosquito physiology and anti-*Plasmodium* defenses has not been examined and requires further investigation.

It has been previously reported that *Plasmodium* infection was associated with significant changes in the expression of miR-34, miR-1174, miR-1175 and miR-989 detected at 24–48 h after *P. berghei* infection [25]. Interestingly, no overlap between sets of differentially expressed miRNAs reported in this study and by Winter et al. [25] was found. This discrepancy most probably reflects the dynamic changes in the miRNA expression profiles after an infectious meal. The further detailed study of the *Plasmodium*-responsive miRNA expression patterns and function may uncover new pathways and effectors that limit the parasite development within its insect host.

## Conclusions

This study provides transcriptional evidences based on the 5p- and 3p-associated read abundance for 123 miRNAs, including distinct 65 miRNAs previously reported in miRBase and 58 newly identified miRNAs in *A. gambiae*. Out of the newly described miRNAs, 21 novel miRNAs are potentially specific to *A. gambiae*. Importantly, sequence read abundance of certain miRNAs, such as newly identified *mir-981*, *mir-33* and other miRNAs (Table 2) was not associated with the mature sequences predicted by sequence similarity. We extended a list of known mosquito-specific miRNAs previously reported by Li et al. [30] by describing 4 novel miRNAs conserved in *Aedes* (Table 1; Additional file 1: Figure S3). Detailed bioinformatics analysis provided evidences for functionally significant variations in mature sequences of *Anopheles* miRNAs and their isoforms occurring at their 5’- and 3’-ends in sugar- and blood-fed mosquitoes (Figure 3). Moreover, we observed substantial variations in relative arm usage and arm-switching events showing the existence of species-specific production of dominant mature miRNAs induced by blood feeding in mosquitoes (Table 2, Figure 4). We identified new conserved and fragmented miRNA clusters and *A. gambiae*-specific miRNA gene duplication (Figure 5). Taken together, sequence variations, functional shifting and switching in mature miRNA sequences, described in this study, diversify significantly miRNA regulatory capacity in *A. gambiae*.

*A. gambiae* mosquitoes are the major vectors of human malaria in sub-Saharan Africa. We identified a set of the differentially expressed miRNAs that early respond to normal and infectious blood meals. The expression levels of the highly abundant miRNAs, miR-7 and exogenous mmu-miR-92a were significantly increased in blood-fed mosquitoes; while miR-317 and miR-2940 were significantly upregulated after *P. berghei* parasite infections. Further experimental study will require to discern the functions of the exogenous miRNAs derived from the host-blood and the *Plasmodium*-responsive miRNAs in the mosquito physiology and immunity.

## Methods

### Sample preparation and small RNA sequencing

*A. gambiae* G3 strain was reared and maintained in humidified chambers at 28°C with a 12 h light/dark cycle. For small RNA sequencing, 4–5 day old female mosquitoes were collected 3 h after a regular and an infectious blood feeding on the anaesthetized CD1 mice. Females fed on 10% sugar solution were used as a control. Infectious blood feeding was performed at 21°C on CD1 mice infected with the *PbGFPCON* strain [51] and fed mosquitoes were kept at 21°C. Two independent biological replicates containing sugar-fed, blood-fed and *P. berghei-*infected females and a single replicate of *Ago1-* and *Ago2-*silenced females were used for small RNA cDNA library preparation. Total RNA was isolated from 30–50 females using a TRI Reagent (MRC). The strand-specific cDNA libraries with different barcodes (6 base index) were generated using a TruSeq Small RNA kit v2 (Illumina). The cDNA libraries were amplified by 13 cycles of polymerase chain reaction (PCR). The final 140–150 nt products were purified and sequenced in the Deep Sequencing facility of the IGBMC using the Illumina sequencing platform. The following adaptors and primers were used for cDNA synthesis and PCR amplification, 3' ligation adapter: *5'–pUCGUAUGCCGUCUUCUGCUUGUidT-3’*; 5' ligation adapter: *5'-GUUCAGAGUUCUACAGUCCGACGAUC-3’*; reverse transcription primer: *5'–CAAGCAGAAGACGGCATACGA-3’*; PCR forward primer: *5'-CAAGCAGAAGACGGCATACGA-3’*; PCR reverse primer: *5'-AATGATACGGCGACCACCGACAGGTTCAGAGTTCTACAGTCCGA-3’*; sequencing primer: *5'-CGACAGGTTCAGAGTTCTACAGTCCGACGATC-3’*.

### Data deposition

Sequencing data have been deposited in the GEO database under the NCBI-GEO accession number GSE50396.

### miRNA identification and prediction

Sequence reads were processed using the CASAVA1.8 pipeline (Illumina). Non-coding RNA profiling was performed by the ncPRO-seq analysis pipeline [52]. miRDeep2 analysis [53] was employed to detect potential miRNAs from raw RNA sequencing data using default parameters. To quantify miR-71 associated sequence reads the following algorithm developed in-house was used: sequence reads mapped to Agamp3 genome assembly, maximum 2 nt mismatches were allowed. Multiple position mapping was enabled and the weight of multiple mapping reads was considered. We allowed 2 nt upstream and downstream shifts in the mapping window for sequenced miR-71-5p and miR-71-3p, if they fall within the same positions on the predicted miRNA-71 precursor. The thermodynamic stability of the secondary structures of the flanking genomic sequences was analyzed using RNAfold and Mfold [54, 55]. IGV2.0 viewer was used to visualize sequence reads mapped to the reference genome. To identify putative homologs, sequence alignment of *A. gambiae* miRNAs with mature miRNAs was performed using *Ae. aegypti* and *D. melanogaster* data sets (miRBase v19) and manually inspected. Mosquito miRNAs showing exact seed matches and sharing more than 70% of sequence similarity were considered as *A. gambiae* homologs of the corresponding known miRNAs. *Anopheles* miRNAs with a low level of sequence similarity were further used for the seed sequence alignment. The alignment was performed using the first 10 nt at the 5’-end sequence of *Anopheles* miRNAs and sequences of known miRNA data sets, including *Ae. aegypti* (AaegL1), *B. mori* (SILKDB2.0), *D. melanogaster* (BDGP5.0), *T. castaneum* (Tcas3.0) and *H. sapiens* (GRCh37.p5). Multiple RNA sequence alignment was performed using the MARNA RNA tool. For quantification of miRNA expression levels, sequence reads with non-template additions at the 3’-end were included in the counts. The DESeq2.6 package was used to quantify and to assess miRNA differential expression, which was considered as significant at *p < 0.05*. The raw log value of all single miRNA reads and the 5p- and 3p-derived reads are listed in Additional file 2: Table S1 and in Additional file 1: Figures S1-2, respectively. Detected *M. musculus* miRNAs in small RNA cDNA libraries are listed in Additional file 3: Table S2. Small RNA expression in *Ago*-silenced mosquitoes was analyzed using miRBase v19, miRDeep2 and Rfam databases. The raw and normalized read frequencies of small RNAs are listed in Additional file 4: Table S3.

### Plasmid construction and RNA-based silencing

RNA interference was used to silence *Ago1* and *Ago2* expression in adult female mosquitoes. *Ago1* (*AGAP011717*): *XhoI-XbaI* 432 bp PCR-amplified fragment was subcloned from the 20AA09 clone of the Gateway (Invitrogen) immune library described in [56] into the pLL110 vector carrying two T7 promoters. *Ago2* (*AGAP011537*): *StuI*-*XhoI* 500 bp fragment was PCR-amplified from a cDNA (*A. gambiae* G3 strain) and cloned into pLL110. The following PCR primers were used, *Ago1* forward primer: *5’-CTGCACCGTTACAGACACG-3’*; *Ago1* reverse primer: *5’-CCAAGTTGCCCCATCCC-3’*; *Ago2* forward primer: *5’-aaaAGGCCTGCCACCGGTAGTGCC-3’*; *Ago2* reverse primer: *5’-ccgctcgagGTTTTCAGCACGCCCAAATC-3’*. Sense and anti-sense single-stranded RNAs were synthesized using MEGAscript T7 kit (Ambion), purified using MEGAclear kit (Ambion) and annealed in an equimolar ratio. One-day post-emerged CO_2_-anaesthetized mosquito females were injected intrathoracically with 0.6 μg of dsRNA using nano-injector (Nanoject II, Drummond). Efficacy of RNA silencing on gene expression was analyzed 24 h after dsRNA injection by quantitative real-time PCR (qRT-PCR).

### qRT-PCR

Efficacy of *Ago1* and *Ago2* silencing in the RNA samples used for RNA sequencing was assessed by SYBR Green-based qPCR (ABI). cDNAs were sythesized from 1 μg of total RNA samples using random primers and RevertAid H Minus cDNA synthesis kit (Fermentas). Ribosomal protein L19 gene (*RplL19*) was used as an internal control to normalize *Ago1* and *Ago2* gene expression. Specific primers were designed using Primer Express 3.0 (ABI): *Ago1* forward primer *5’-ACGATGCGGCGCAAGTAT-3’*; *Ago1* reverse primer *5’-CGGGAAGGATTGCATTTGTG-3’*; *Ago2* forward primer *5’-ATGCTCAAGATCAACGCCAAA-3’*; *Ago2* reverse primer *5’-TGAGCGGGTGCGTAACGT-3’*; *RpL19* forward primer *5’-CCAACTCGCGACAAAACATTC-3’*; *RpL19* reverse primer *5’- ACCGGCTTCTTGATGATCAGA-3’*. TaqMan qRT-PCR based quantification of miR-989 and 5.8S rRNA expression levels were performed using TaqMan miRNA RT kit and custom TaqMan small RNA assays (ABI). Relative miR-989 levels were normalized to 5.8S rRNA. 10 ng of total RNA were used for cDNA synthesis. The RT reaction without reverse transcriptase was used as a negative control. PCR reactions were performed on an OneStep Plus thermocycler (ABI), according to the manufacturer’s protocol, and each measurement was derived from three independent biological replicates. Relative quantification of gene expression was performed using the comparative Ct (ΔΔCt) method.

### miRNA sequence variations and preferred arm usage

Analyses of the miRNA sequence variations and the relative arm usage were done using an algorithm developed in-house. The quantification was executed with a pipeline of custom developed Python scripts available upon request. The most abundant sequence reads were used as a reference. No mismatch was allowed within mature miRNA sequences. To identify template-directed variations, sequence reads with 3 nt and less difference at the 5’- and 3’-ends from the most frequently sequenced reads derived from both arms were retrieved and quantified. To characterize non-template directed nucleotide additions, sequence reads with single nucleotide addition mismatched at the 3’-end were quantified. Sequence compilations for specific mature miRNAs were generated using WebLogo3.3. The 5’- and 3’-arm usage of miRNA hairpins was quantified as a proportion of the 5p-associated reads with respect to the total number of reads from miRNAs in *A. gambiae*. Published small RNA data sets for *D. melanogaster* [34] and *Ae. aegypti* female gut-specific libraries [30] were used for the relative arm usage analyses. The relative arm usage was calculated as a ratio of the 5’- to the 3’-arm associated reads of the hairpin precursor and shown as log values. To identify *A. gambiae* miRNA clusters, a 1 kb, 5 kb, 10 kb and 50 kb cut-off were used. Cluster conservation analyses between *A. gambiae* and *Ae. aegypti* was performed using 10 kb cut-off for genomic distance.

### Ethics statement

All vertebrate animals were housed and handled in accordance with the animal protection law (§8 Tierschutzgesetz) and both institutional (Max Planck Society) and national (Landesamt für Gesundheit und Soziales (LAGeSo) regulations. All experimental procedures on mice were approved by the committee for animal use and protection (LAGeSo permit number: H 0027/12).

## Electronic supplementary material

Additional file 1: Figure S1-S2: Containing expression profiles of known *A. gambiae* miRNAs (miRBase v19). **Figure S3.** Containing predicted secondary structures of the putative novel miRNA hairpins conserved in mosquito species. **Figure S4.** Containing summary of the 5’- and 3’-end sequence heterogeneity of mature and star miRNA sequence reads in sugar and blood-fed mosquitoes. **Figure S5.** Containing transcriptional evidences supporting new paralogous members of *mir-276, mir-286* and *mir-309* genes. (PDF 1 MB)

Additional file 2: Table S1: Containing *A. gambiae* miRNA counts from small RNA sequencing of sugar-fed, blood-fed and *P. berghei* infected mosquitoes. (XLSX 40 KB)

Additional file 3: Table S2: Containing *M. musculus* miRNA counts detected in mosquito small RNA cDNA libraries. (XLSX 236 KB)

Additional file 4: Table S3: Containing quantification of the *A. gambiae* small RNA expression in *Ago*-silenced mosquitoes. (XLSX 65 KB)
