# Supplementary material for: Transcriptome-wide analysis of microRNA expression in the malaria mosquito Anopheles gambiae
Source: BMC Genomics. 2014 Jul 4;15(1):557. doi: 10.1186/1471-2164-15-557 (PMC4112208; doi:10.1186/1471-2164-15-557)
Supplement: Supplementary file 1 — Additional file 1: Figure S1-S2: Containing expression profiles of known A. gambiae miRNAs (miRBase v19). Figure S3. Containing predicted secondary structures of the putative novel miRNA hairpins conserved in mosquito species. Figure S4. Containing summary of the 5’- and 3’-end sequence heterogeneity of mature and star miRNA sequence reads in sugar and blood-fed mosquitoes. Figure S5. Containing transcriptional evidences supporting new paralogous members of mir-276, mir-286 and mir-309 genes. (PDF 1 MB) [file 12864_2013_6282_MOESM1_ESM.pdf]

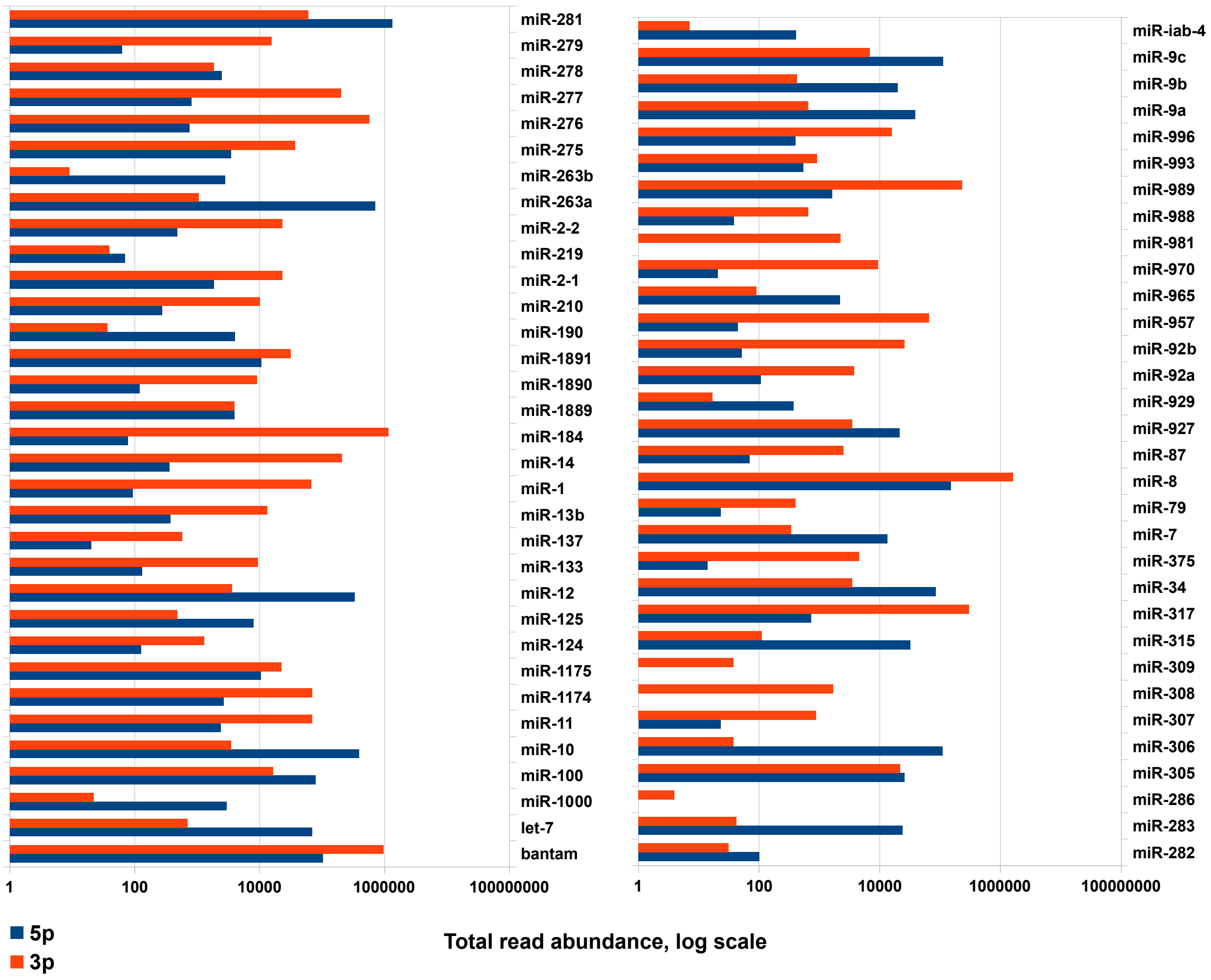

Additional figure S1

Expression profiles of known *A. gambiae* miRNAs cataloged in miRBase v19. The frequency of reads supporting the 5p- (blue) and 3p-sequences (red) from sugar-fed mosquitoes is as indicated.

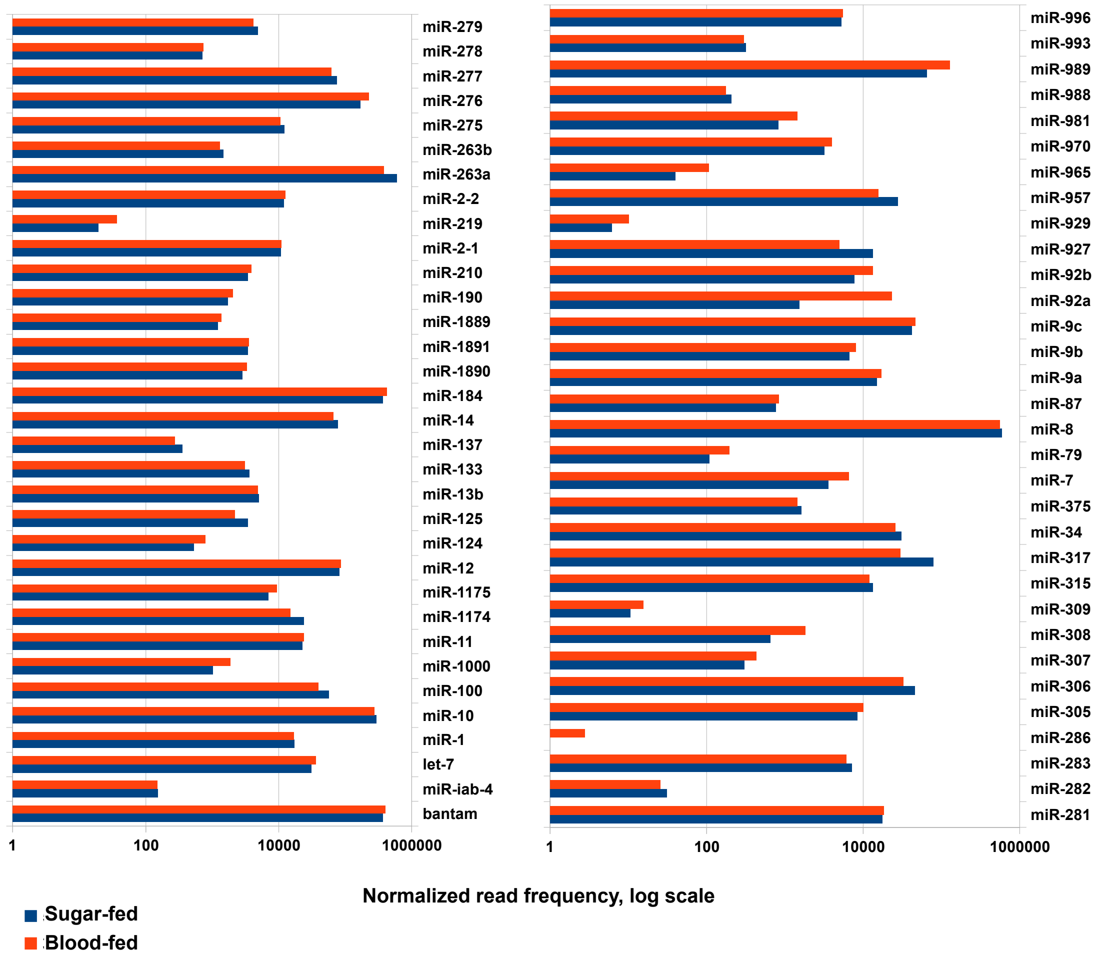

Additional figure S2

The normalized mature miRNA (miRBase) read frequencies in sugar (blue) and blood-fed (red) mosquitoes.

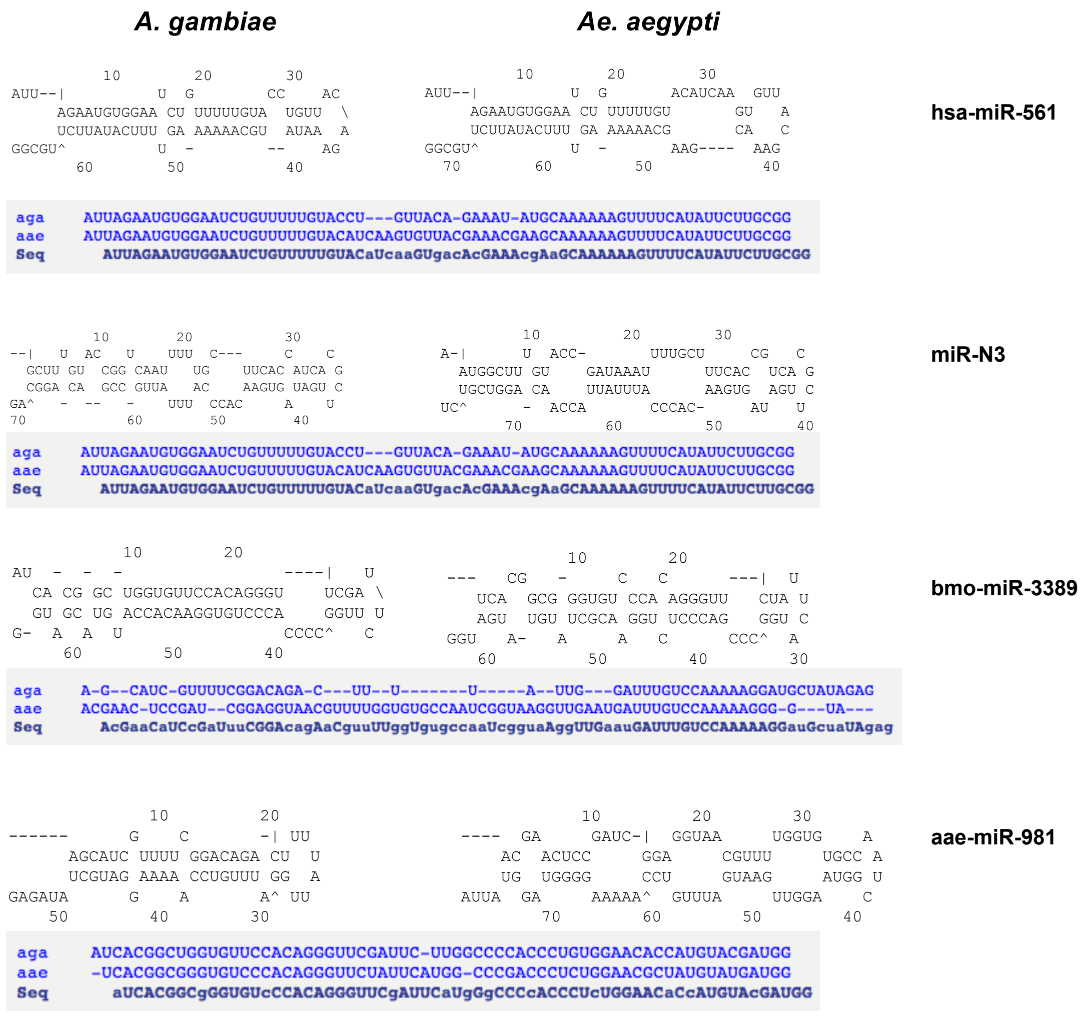

Additional figure S3

Predicted secondary structures of the putative novel miRNA hairpins conserved in mosquito species; sequence alignments of putative orthologous *Aedes* miRNA precursors are shown below.

hsa - *H. sapiens*

bmo - B. mori

aae - Ae. aegypti

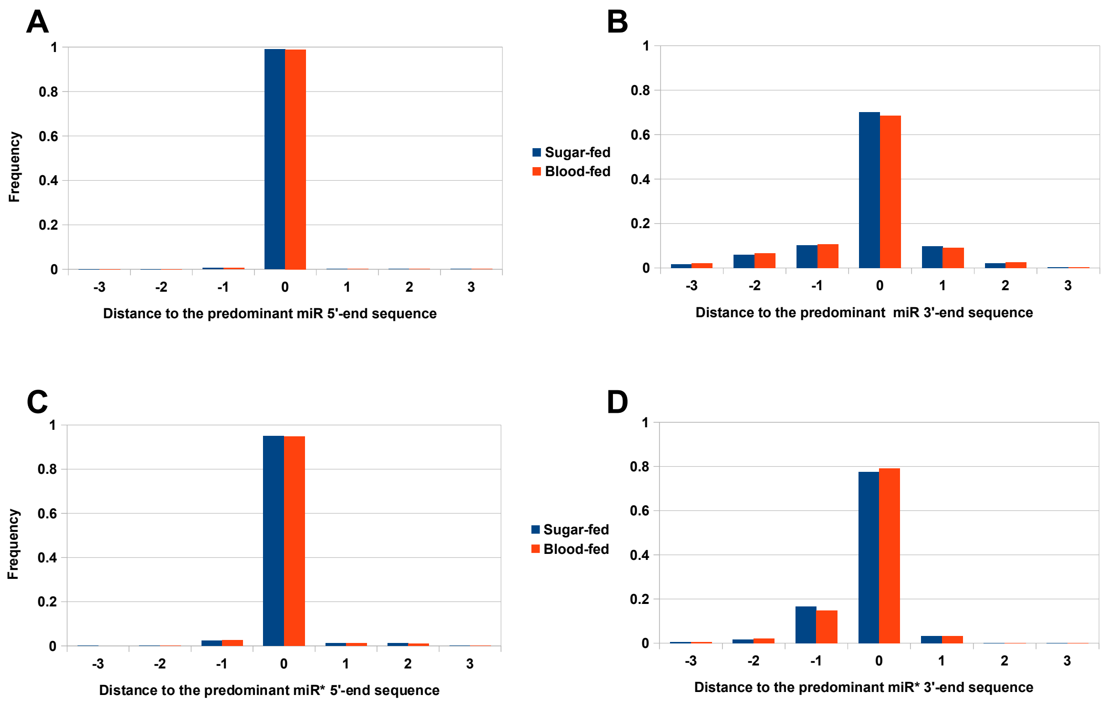

Additional figure S4

5'- and 3'-end sequence heterogeneity of mature (A-B) and star (C-D) miRNA sequence reads in sugar (blue) and blood-fed (red) mosquitoes as indicated.

aga-*mir-276-1* chr2L:18991754-18991829

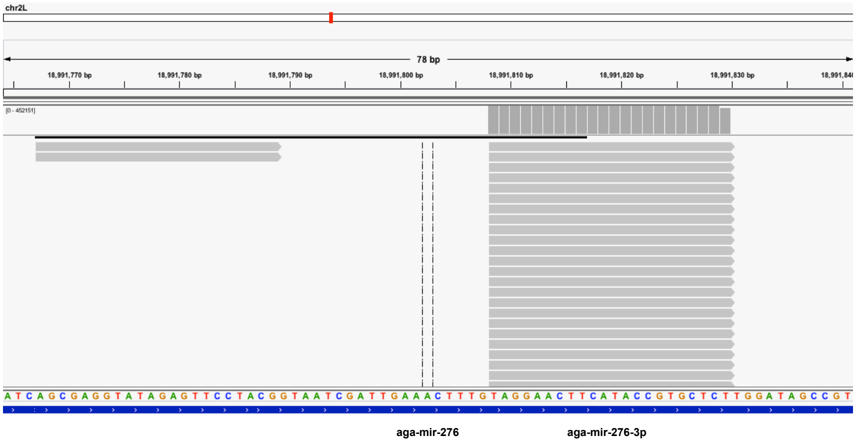

mir-276-2 chr2L:18921593-18921565

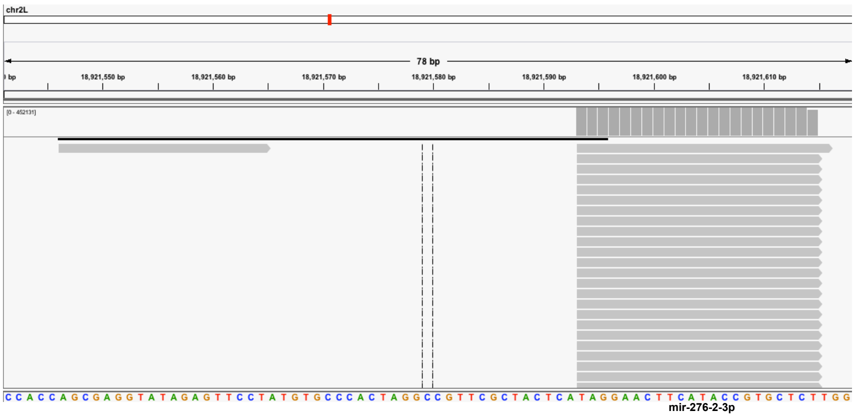


```
mir-276-1     GGUGACUGCCAUCAGCGAGGUAUAGAGUUCCUACGGU--AAUCGAUU--GAA----ACUUUGUAGGAACUUCAUACCGUGCUCUUGGAUAGCC
mir-276-2     -----C--------AGCGAGGUAUAGAGUUCCUA-UGUGCCCACUAGGCCGUUCGCUACUCA-UAGGAACUUCAUACCGUGCUCU----U----
Seq           ggugaCugccaucAGCGAGGUAUAGAGUUCCUAcgGUgcaaaCgAggccGaacgcuACUcagUAGGAACUUCAUACCGUGCUCUuggaUagcc

mir-276-1     (((.....(((..((((.(((((..(((((((((((.--..((....--)).----...)))))))))))..))))).))))..)))...)))
mir-276-2     -----.-------((((.(((((..((((((((-((.((..((......))..))....))-))))))))..))))).))))..----.----
```

aga-*mir-309-1* chr3R:43008784-43008711

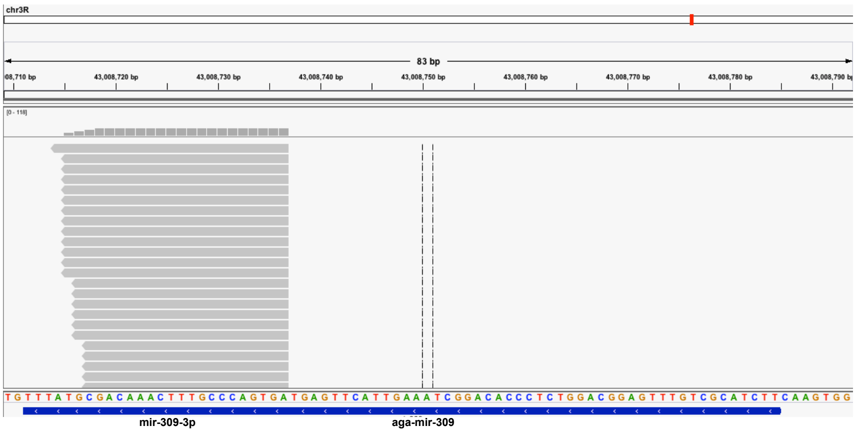

mir-309-2 2L:3536967-3536898

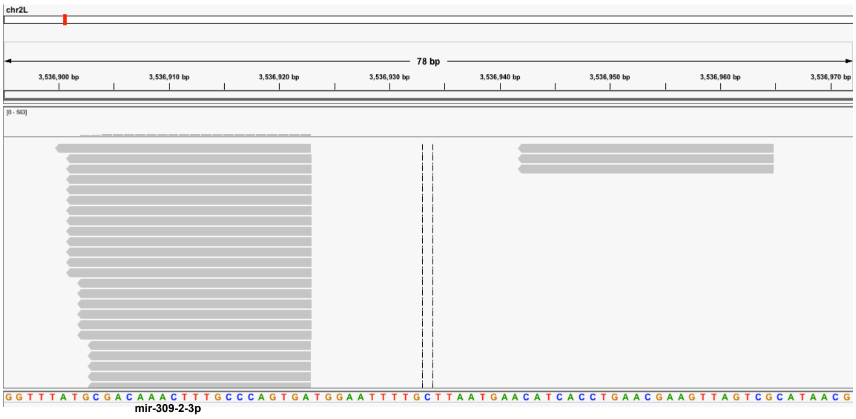

```
mir-309-1     AAGAUGCGACAAACUCCGUCCAGAGGGUGUCCGAUUUCAAUGAA-CUCAUCACUGGGCAAAGUUUGUCGCAUAAA
mir-309-2     ---AUGCGACUAACUUCGUUCAGGUGAUGUUC-AUUAAGCAAAAUUCCAUCACUGGGCAAAGUUUGUCGCAUAA-
Seq           aagAUGCGACaAACUcCGUcCAGagGaUGUcCgAUUaaaaaaAAuccCAUCACUGGGCAAAGUUUGUCGCAUAAa

mir-309-1     ...((((((((((((..((((((..((((...............-..)))).))))))..))))))))))))...
mir-309-2     ---(((((((.(((((.((((((.(((((...-(((......)))..))))))))))).))))).))))))).-
```

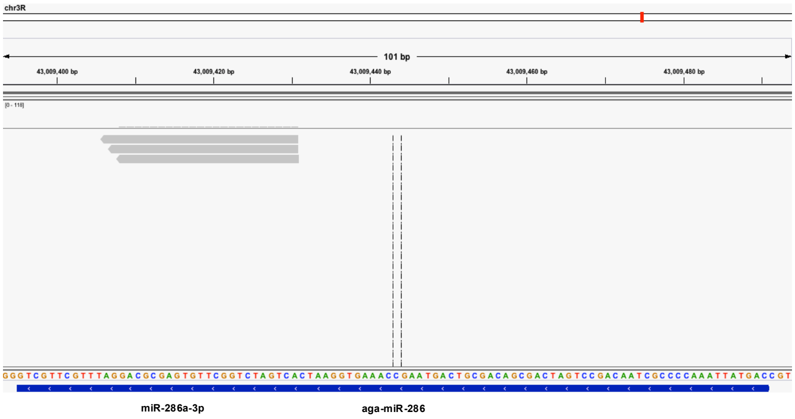

aga-*mir-286a* chr3R:43009395-43009490

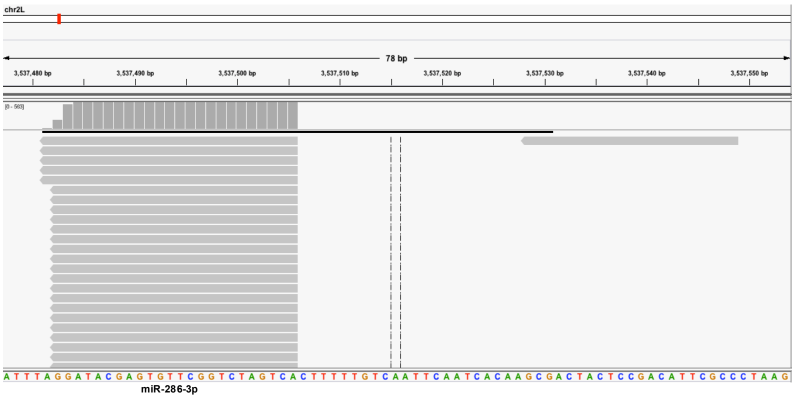

mir-286b chr2L:3537482-3537547

```
mir-286a     GUCAUAAUUUGGGGCGAUUGUCGGACUAGUCGCUGUCG-CAGUCAUUCGGUUU-CACCUUAGUGACUAGACCGAACACUCGCGUCCUAAACGAA
mir-286b     -------------GCGAAUGUCGGAGUAGUCGCU-U-GUGA-U--UGA-AUUGACAAAA-AGUGACUAGACCGAACACU--CGU--------AU
Seq          gucauaauuugggGCGAaUGUCGGAcUAGUCGCUgUcGucAgUcaUgagaUUgaCAaaauAGUGACUAGACCGAACACUcgCGUccuaaacgAa

mir-286a     (((....(((((((((..((((((.((((((((((..(-.(..(....)..).-)....)))))))))).))).))).....)))))))))....
mir-286b     -------------((((.((((((..((((((((-(-((.(-.--...-..).)))...-))))))))..))).))).)--)))--------..
```

Additional figure S5

Transcriptional evidences supporting new paralogous members of *mir-276, mir-286* and *mir-309* genes (miRBase). Sequence alignments of paralogous miRNA precursors are shown below.
